# The Effects of Bilateral and Ipsilateral Auditory Stimuli on the Subcomponents of Visual Attention

**DOI:** 10.1177/20416695211058222

**Published:** 2021-12-23

**Authors:** Jing Fu, Xuanru Guo, Xiaoyu Tang, Aijun Wang, Ming Zhang, Yulin Gao, Takeharu Seno

**Affiliations:** School of Psychology, Liaoning Collaborative Innovation Center of Children and Adolescents Healthy Personality Assessment and Cultivation, 66523 Liaoning Normal University, Dalian, China; Faculty of Design, 12923 Kyushu University, Minami-ku, Fukuoka, Japan; School of Psychology, Liaoning Collaborative Innovation Center of Children and Adolescents Healthy Personality Assessment and Cultivation, 66523 Liaoning Normal University, Dalian, China; Department of Psychology, 12582 Soochow University, Suzhou, China; Department of Psychology, 12510 Jilin University, Changchun, China; Faculty of Design, 12923 Kyushu University, Minami-ku, Fukuoka, Japan

**Keywords:** attention networks, alerting, orienting, executive control, bilateral or ipsilateral auditory stimulus, relative multisensory response enhancement

## Abstract

Attention contains three functional network subcomponents of alerting, orienting, and executive control. The attention network test (ANT) is usually used to measure the efficiency of three attention subcomponents. Previous researches have focused on examining the unimodal attention with visual or auditory ANT paradigms. However, it is still unclear how an auditory stimulus influences the visual attention networks. This study investigated the effects of bilateral auditory stimuli (Experiment 1) and ipsilateral auditory stimulus (Experiment 2) on the visual attention subcomponents. We employed an ANT paradigm and manipulated the target modality types, including visual and audiovisual modalities. The participants were instructed to distinguish the direction of the central arrow surrounded by distractor arrows. In Experiment 1, we found that the simultaneous bilateral auditory stimuli reduced the efficiency of visual alerting and orienting, but had no significant effect on the efficiency of visual executive control. In Experiment 2, the ipsilateral auditory stimulus reduced the efficiency of visual executive control, but had no significant effect on the efficiency of visual alerting and orienting. We also observed a reduced relative multisensory response enhancement (rMRE) effect in cue condition relative to no cue condition (Experiment 1), and an increased rMRE effect in congruent condition compared with incongruent condition (Experiment 2). These results firstly provide evidence for the alerting, orienting and executive control effects in audiovisual condition. And the bilateral and ipsilateral auditory stimuli have different effects on the subcomponents of visual attention.

## Introduction

At every moment, we are bombarded with an overpowering amount of sensory information (e.g., sights, sounds, tactile sensations, tastes and so on). Attention plays a main role in the cognitive processes of human as a mechanism that enables the selection of stimuli from a multitude of sensory information, and helps the brain integrate useful stimuli into coherent cognition (Giard & Peronnet, 1999). It is suggested that attention contains three functionally interrelated but anatomically separate attention subcomponents: alerting network, orienting network and executive control network (Petersen & Posner, 2012; Posner & Petersen, 1990). Alerting network is responsible for providing and maintaining the vigilance to a forthcoming stimulus. Orienting network is responsible for selecting specific information among an overwhelm amount of sensory input. Executive control network involves more complicated mental mechanism that is responsible for inhibiting competitive information and resolving conflicts.[Table table1-20416695211058222][Table table2-20416695211058222]

**Table 1. table1-20416695211058222:** Average of reaction times (RTs; SD) in milliseconds and accuracy (ACC; SD) in percentage, for all combinations of modality type (V, AV), cue type (no cue, double cue, center cue, and spatial cue) and flanker type (neutral, congruent, and incongruent) in Experiment 1.

Cue type	Flanker type	Modality type			
		V		AV	
		RT (ms)	ACC (%)	RT (ms)	ACC (%)
No cue	Neutral	532.10(50.59)	98.65(2.64)	513.26(51.04)	98.72(2.27)
	Congruent	531.34(55.76)	99.08(1.97)	512.67(55.16)	99.50(1.16)
	Incongruent	576.87(61.88)	97.44(3.03)	564.70(50.63)	94.96(4.96)
Double cue	Neutral	492.24(52.42)	98.22(2.37)	484.32(51.29)	98.65(2.17)
	Congruent	487.42(55.38)	99.08(1.97)	476.54(51.42)	99.43(1.22)
	Incongruent	550.53(57.09)	93.82(5.93)	540.22(59.97)	94.32(5.85)
Center cue	Neutral	491.07(55.25)	98.58(1.84)	479.12(50.40)	98.58(2.28)
	Congruent	486.94(54.05)	98.93(1.78)	481.03(55.97)	98.44(2.57)
	Incongruent	562.20(62.85)	91.48(7.52)	548.43(58.11)	91.41(6.84)
Spatial cue	Neutral	460.61(50.74)	98.30(2.38)	452.20(52.80)	98.51(2.38)
	Congruent	454.79(51.19)	99.50(1.50)	459.38(50.39)	99.01(2.00)
	Incongruent	511.89(69.37)	96.45(4.45)	502.12(63.51)	95.24(5.15)

*Note*. V: visual modality, AV: audiovisual modality.

**Table 2. table2-20416695211058222:** Average of reaction times (RTs; SD) in milliseconds and accuracy (ACC; SD) in percentage, for all combinations of modality type (V, AV), cue type (no cue, double cue, center cue, and spatial cue) and flanker type (neutral, congruent, and incongruent) in experiment 2.

Cue type	Flanker type	Modality type			
		V		AV	
		RT (ms)	ACC (%)	RT (ms)	ACC (%)
No cue	Neutral	508.40(50.66)	98.60(2.83)	445.81(63.07)	99.47(1.19)
	Congruent	506.47(49.25)	99.67(1.17)	442.52(63.65)	99.73(0.88)
	Incongruent	558.54(58.73)	95.68(4.63)	520.98(73.52)	95.74(5.02)
Double cue	Neutral	489.89(56.41)	99.07(1.83)	434.35(71.75)	99.07(2.05)
	Congruent	486.01(51.83)	99.20(1.66)	431.83(71.23)	99.47(1.19)
	Incongruent	551.39(56.94)	95.61(4.99)	502.89(86.00)	97.21(4.38)
Center cue	Neutral	487.40(53.89)	98.80(2.40)	435.98(69.91)	98.74(2.13)
	Congruent	489.87(61.14)	99.27(1.49)	432.68(69.20)	99.67(1.17)
	Incongruent	548.97(54.39)	93.28(8.16)	501.44(91.16)	95.48(5.64)
Spatial cue	Neutral	471.04(54.88)	99.20(1.66)	421.51(68.50)	98.74(2.13)
	Congruent	468.39(54.90)	99.27(1.62)	420.01(64.78)	99.67(1.17)
	Incongruent	518.67(59.28)	97.01(3.96)	476.22(82.15)	95.48(5.64)

*Note.* V: visual modality, AV: audiovisual modality.

One classic procedure for simultaneously studying these three attention subcomponents is the attention network test (ANT) (Fan et al., 2002), which combined the spatial cueing paradigm (Posner, 1980) with a flanker task (Eriksen & Eriksen, 1974). The visual version of attention network test, introduced by Fan et al. (2002), has revealed how to calculate the efficiency of alerting, orienting and executive control networks. Since then, numerous researches have used visual attention network test (vANT) or auditory attention network test (aANT) to examine unimodal visual or auditory attention (Johnston et al., 2019; Macleod et al., 2010). Yet, in ANT paradigm, it is still unclear the effect of auditory stimuli on the visual attention networks.

Despite limited evidence in ANT, studies using other attention tests found that auditory stimuli could affect the alerting, orienting, or executive control network within the visual domain, respectively. Several findings have revealed that the response to multisensory (e.g., audiovisual) target could be faster and more accurate than response to unisensory (e.g., visual or auditory) target, that is, the redundant signals effect (Hershenson, 1962; Talsma & Woldorff, 2005; Tang et al., 2016). It has been found that the orienting effect was enhanced when the synchronized auditory stimuli and visual target were presented at the same location (Frassinetti et al., 2002; Ngo & Spence, 2010). Further on, Gao et al. (2014) designed a visual detection task to examine the influence on the orienting effect of visual target with two types of auditory stimuli. In their study, the visual target was presented simultaneously with ipsilateral auditory stimulus or bilateral auditory stimuli. The behavioral results showed that, compared with unimodal visual condition, the visual orienting effect could be enhanced by both ipsilateral auditory stimulus and bilateral auditory stimuli. The event-related potential (ERP) data found that the audiovisual integration effect induced by the visual stimuli and the bilateral auditory stimuli differed from that induced by the visual stimuli and the ipsilateral auditory stimulus. The audiovisual integration of early-sensory and late-cognitive processing stages were observed in the ipsilateral auditory condition, while the integration in the bilateral auditory condition only occurred at the late stages of cognitive processing. The results of differences between bilateral and ipsilateral auditory conditions are suggested to correlate with the spatial principle of auditory integration, for which the spatial coincidence (different sensory input was presented from the same location) was deemed to further facilitate the audiovisual integration effect (Stein & Meredith, 1990). Since bilateral and ipsilateral auditory stimuli elicited different integration effect, they may influence differently on the subcomponents of visual attention. Therefore, we manipulated bilateral and ipsilateral auditory stimuli in ANT to investigate the effects of auditory stimuli on the visual attention subcomponents.

In the domain of executive control, previous studies have indicated that synchronized auditory stimuli might affect conflict resolving (Guo et al., 2015; Lacey et al., 2020; Spilcke-Liss et al., 2019). Laurienti and his colleagues examined the effect of semantic stimulus congruence on multisensory target (i.e., paired visual-auditory) performance, compared with unimodal target (i.e., visual or auditory) performance. They found that response to the congruent multisensory target was significantly faster than response to the unimodal target, while the incongruent multisensory target was slower than the unimodal target (Laurienti et al., 2004). This “congruency effect”, suggesting that semantically consistent multisensory stimuli helped conflict resolving. However, does the spatial consistency of multisensory stimuli affect the resolution of spatial conflict tasks? Fong et al. (2018) designed a Go/No-go flanker task to examine whether the cross-modal auditory stimuli that were aligned with the flanker direction affected conflict resolving. The results showed that the ipsilateral auditory stimulus (in the same direction as the flanker arrow) may cause additional conflicts, resulting in reducing executive control efficiency (Fong et al., 2018). In the ANT paradigm, we speculated that the spatial information provided by ipsilateral auditory stimulus may affect the identification of the central arrow direction, but there is no evidence suggest that bilateral auditory stimuli have an effect on this.

According to the above, the role of auditory stimuli on the visual attention networks is complex and multifaceted. Nevertheless, in ANT paradigm, we are aware of only one research (Mahoney et al., 2012) involving the effects of synchronized auditory stimuli on visual attention subcomponents, in which multisensory stimuli were manipulated as cues. Mahoney and his colleagues designed a modified version of ANT, where unisensory cues (V: visual; A: auditory; and S: somatosensory) and multisensory cues (AV: auditory-visual; AS: auditory-somatosensory; and VS: visual-somatosensory) were added. The results showed that only youth participants demonstrated both significant alerting effect and orienting effect across AV cue. However, it remains open whether the synchronized auditory stimuli manipulated as targets affected the visual attention subcomponents in ANT paradigm.

The present study aims to examining the effect of auditory stimuli on the alerting, orienting and executive control networks of visual attention. Based on previous studies in which both bilateral and ipsilateral auditory stimuli could affect the orienting effect, while the executive control effect was only affected by ipsilateral auditory stimulus, we manipulated the sounds position. In Experiment 1, the visual target was presented simultaneously with bilateral auditory stimuli, which were presented by two peripheral speakers on the left and right of the screen. In Experiment 2, the visual target was presented simultaneously with ipsilateral auditory stimulus, which was presented by one peripheral speaker on the left or right side of the screen.

In this task, the audiovisual stimuli consisted of visual stimuli accompanied by simultaneous bilateral or ipsilateral auditory stimuli. We manipulated the cue type (no cue, double cue, center cue, and spatial cue), flanker type (neutral, congruent, and incongruent) and modality type (visual, audiovisual), and compared the mean reaction times (RTs) and accuracy of the targets in the visual condition to the audiovisual condition. In accordance with previous researches, we suggested that the bilateral and ipsilateral auditory stimuli have different effects on the visual attention subcomponents. Specifically, based on the similar enhanced orienting effect has been found in visual detection tasks (Gao et al., 2014; Tang et al., 2020), we predicted that the alerting and orienting effects of visual attention may be affected by bilateral auditory stimuli as well as ipsilateral auditory stimuli. Based on the similar reduced executive control effect has been found in flanker tasks (Fong et al., 2018), we predicted that the executive control effect of visual attention may be affected by ipsilateral auditory stimulus (in the same direction as the target arrow), but not bilateral auditory stimuli.

## Experiment 1

### Materials and Methods

#### Participants

Forty-four undergraduate University students aged 19–24 years (39 females, *M* = 20.56 years, *SD* = 1.43) volunteered to participate in this study. Using the G*Power toolbox (Faul et al., 2007) to calculate the sample size, to detect the corresponding effect size of f value (α = 0.05; power = 0.80), a sample of 24–48 participants was required for the Experiment 1. All participants reported normal or corrected-to-normal vision, no hearing impairment, and no history of neurological or psychiatric disorders. Before the experiment, all participants gave written informed consent. The experimental protocol was reviewed and approved by the Ethics Committee of Liaoning Normal University.

#### Apparatus and Stimuli

The experiment was conducted in a dimly lit, sound-attenuated room. An HP ProBook 440 G4 LCD (14-inch) display equipped with a screen resolution of 1366 × 768 pixels, and a refresh rate of 60 Hz. Visual stimuli were presented on a gray background screen (RGB:127,127,127) 65 cm from the subjects. Auditory stimuli were presented via two speakers placed at both the left and the right of the screen (hidden behind the screen). MATLAB 2013b (Mathworks, Natick, MA) and PsychToolbox-3 (Brainard, 1997) were used to control the presentation of stimuli and the acquisition of data.

The fixation stimulus was a black cross (RGB: 255, 255, 255; length: 0.05° × 0.05°), displayed at the central location of screen. One of four different types of the cue prior to the target was presented: (1) no cue: no warning stimulus was presented prior to the target; (2) double cue: two asterisks appeared above and below the fixation cross; (3) center cue: an asterisk briefly replaced the fixation cross; (4) spatial cue: an asterisk appeared above or below the central fixation cross. The spatial cue could accurately predict the location of target. The visual stimuli consisted of a row of five horizontal black arrows, or a central arrow flanked with the left and right by two horizontal black lines, with arrowheads pointing leftward or rightward. The central arrow (length: 1.6°; away from the center fixation: 0.6°) was the target, and the other arrows or lines were flankers. The central arrow pointed to left or right, and the flanker arrows pointed in the same direction as the former (congruent condition); the central arrow pointed to left or right, while the flanker arrows pointed in the opposite direction (incongruent condition); or the central arrow pointed to left or right, which was flanked by horizontal black lines (neutral condition). The arrows appeared on above or below the fixed cross with equal probability.

The auditory stimuli were 500 Hz pure tone (60 ms), which were presented simultaneously by the left and right speakers. The modality types could be unimodal visual (V; without sound) or audiovisual (AV; with sound), shown in random with equal probability.

#### Procedure and Design

**Figure 1.**
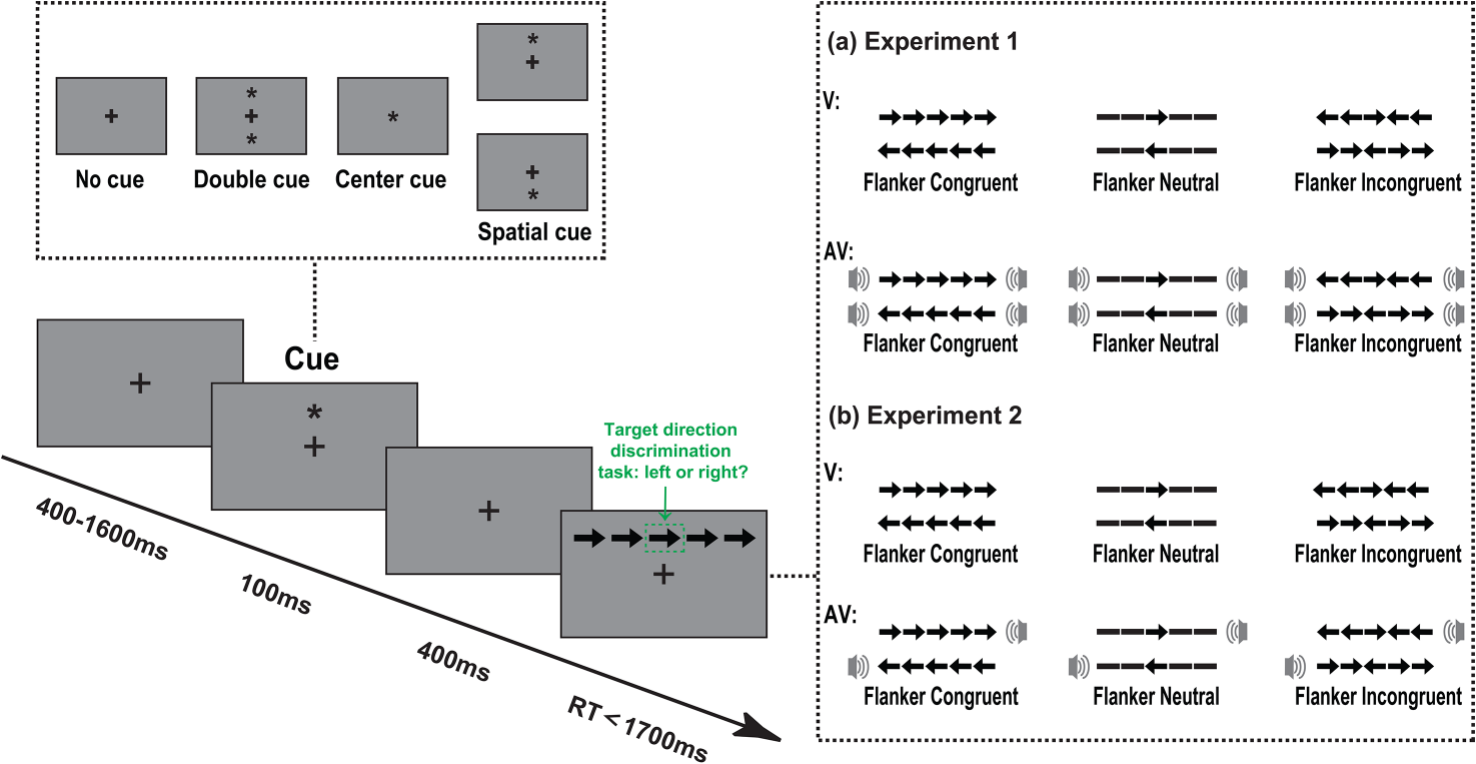
Schematic representation of the experimental procedures in two experiments. (a) The arrow conditions in Experiment 1. (b) The arrow conditions in Experiment 2. The participants were instructed to make a response to the direction of the target arrow (central arrow), which surrounded by two flanker arrows or two lines (neutral condition). The two flanker arrows on each side pointing either to the same direction as the target (congruent condition) or to the opposite direction (incongruent condition).

The experiment manipulated three factors of cue type (no cue, center cue, double cue, and spatial cue), flanker type (neutral, congruent, and incongruent) and modality type (V, AV). Each trial started with the presentation of a fixation cross with a random duration between 400 and 1600 ms (see Figure 1). Following the fixation stimulus, one of the four cues was presented for 100 ms. After a fixed cue-target interval of 400 ms, the target stimuli were presented and maintained until a response made by participant or until response times exceeded 1700 ms. Participants were instructed to ignore flankers and identify the direction (left or right) of the central arrow as quickly and accurately as possible, using two keys (“F” for the left and “J” for the right) on the keyboard. Each experimental run consisted of 8 blocks, and each block consisted of 96 trials. Participants practiced in 20 trials before the formal experiment began. Participants were shown totally 768 trials during the experimental phase and were allowed to take a break between blocks. The entire experiment lasted for about 40 minutes.

#### Data Analysis

Response accuracy and reaction times were collected as experimental data. All missing or false trials were removed from the data. Outliers were defined as three standard deviations (SD) above or below the mean reaction times, and were removed from the experimental data. The final data deletion accounted for 3.89% of the total data. A 4 (cue type: no cue, double cue, center cue, and spatial cue) × 3 (flanker type: neutral, congruent, and incongruent) × 2 (modality type: V, AV) repeated-measures ANOVA was conducted on the accuracy and reaction times. The Greenhouse–Geisser epsilon correction was used to correct for non-sphericity. The Bonferroni correction was applied to the post-hoc comparisons. The effect size of the partial eta squared (η_p_^2^) was calculated for the ANOVA.

Using mean reaction time (RT), a set of cognitive subtractions (described below) was used to assess the efficiency of three attention networks (Fan et al., 2002):

Alerting effect = RT (no cue) – RT (double cue)

Orienting effect = RT (center cue) – RT (spatial cue)

Executive control effect = RT (incongruent) – RT (congruent)

Median response times of each participant in each condition were used for further analyses. The relative multisensory responses enhancement (rMRE) value reflects the relative amount of acceleration or deceleration of the individual when responding to a multisensory modalities target as compared to when responding to a unimodal target. In Experiment 1, we asked participants to respond to V or AV, but did not set unimodal auditory trials. Thus, we calculated the rMRE using a single visual condition (Tang et al., 2019). The Cohen's *d* was used to report the effect sizes and all statistical levels (i.e., α level) were set to 0.05.

$$\mathrm{rMRE}=\frac{\mathrm{median}(\mathrm{RT}{}_{V})\;\text{–}\;\mathrm{median}(\mathrm{RT}{}_{A}{}_{V})}{\mathrm{median}(\mathrm{RT}{}_{V})}\;\times \;100\text{\%}$$

### Results

#### Accuracy

Accuracy was high with participants completing above 90% of trials correctly overall. A repeated 4 × 3 × 2 ANOVA with cue type (no cue, double cue, center cue, and spatial cue), flanker type (neutral, congruent, and incongruent) and modality type (V, AV) was conducted on accuracy. There was a main effect of cue type [*F* (3, 129) = 16.819, *p* < 0.001, η_p_^2^ = 0.281], which was driven by participants responding more accurately to no cue (98.06%) than center cue (96.24%, *p* < 0.001) or double cue (97.25%, *p* = 0.038). The main effect of flanker type was significant [*F* (2, 86) = 67.778, *p* < 0.001, η_p_^2^ = 0.612], caused by participants responding more accurately to congruent condition (99.12%) than neutral condition (98.53%, *p* < 0.001) or incongruent condition (94.39%, *p* < 0.001). There was no main effect of modality type [*F* (1, 43) = 1.704, *p* = 0.199, η_p_^2^ = 0.038]. A significant modality type and flanker type interaction [*F* (2, 86) = 3.486, *p* = 0.043, η_p_^2^ = 0.075] and a significant cue type and flanker type interaction [*F* (6, 258) = 13.085, *p* < 0.001, η_p_^2^ = 0.233] were revealed. No significant interaction was found in modality type and cue type [*F* (3, 129) = 2.264, *p* *=* 0.084, η_p_^2^ = 0.050]. The three-way interaction between modality type, cue type, and flanker type was not significant [*F* (6, 258) = 2.039, *p* *=* 0.087, η_p_^2^ = 0.045].

#### Reaction Time

A repeated 4 × 3 × 2 ANOVA with cue type (no cue, double cue, center cue, and spatial cue), flanker type (neutral, congruent, and incongruent), and modality type (V, AV) was conducted on reaction times. There was a main effect of cue type [*F* (3, 129) = 246.344, *p* < 0.001, η_p_^2^ = 0.851], indicating that participants responded more slowly in no cue condition (538.49 ms) than in double cue (505.21 ms, *p* < 0.001), center cue (508.13 ms, *p* < 0.001), and spatial cue conditions (473.50 ms, *p* < 0.001). In addition, participants responded more slowly in both double cue and center cue conditions than in spatial cue condition (center cue vs. spatial cue, *p* < 0.001; double cue vs. spatial cue, *p* < 0.001). There was a main effect of flanker type [*F* (2, 86) = 427.631, *p* < 0.001, η_p_^2^ = 0.909], caused by participants responding more slowly in the case of incongruent condition (544.62 ms) than in the case of neutral (488.12 ms, *p* < 0.001) and congruent conditions (486.26 ms, *p* < 0.001). The main effect of modality type was significant [*F* (1, 43) = 99.436, *p* < 0.001, η_p_^2^ = 0.698], which was driven by participants responding more slowly to visual modality than audiovisual modality (511.50 ms vs. 501.17 ms).

The interaction between modality type and cue type was significant [*F* (3, 129) = 13.075, *p* < 0.001, η_p_^2^ = 0.233], which could be explained by differences in the sizes of cue effects for different modalities. There was also a significant interaction between modality type and flanker type [*F* (2, 86) = 3.254, *p* = 0.043, η_p_^2^ = 0.070], which could be explained by differences in the sizes of flanker effects for different modalities. A significant cue type and flanker type interaction [*F* (6, 258) = 18.897, *p* < 0.001, η_p_^2^ = 0.305] was revealed, and a three-way interaction between cue type, flanker type, and modality type was significant [*F* (6, 258) = 3.862, *p* = 0.003, η_p_^2^ = 0.082].

#### Attention Network Effects

We reported the difference of attention network effects between visual and audiovisual modalities using repeated-measures ANOVA.

##### The alerting effect

**Figure 2.**
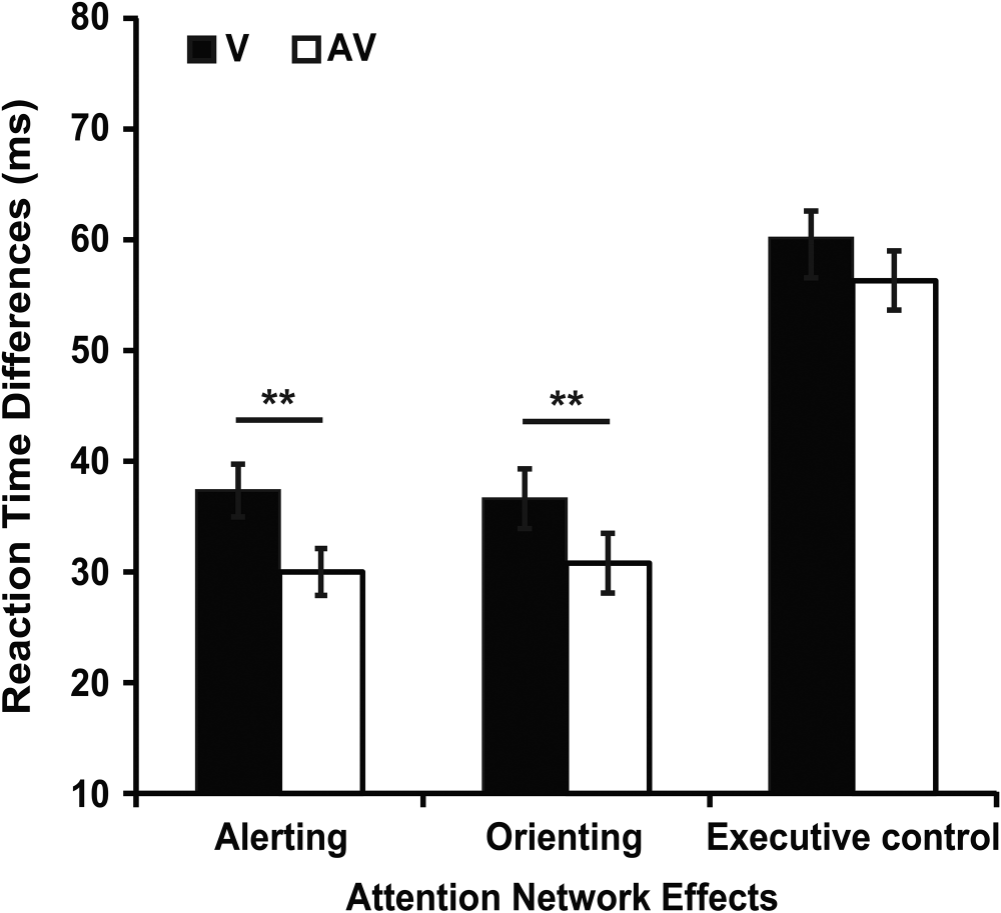
The reaction time differences of attention network effects between visual and audiovisual modalities in Experiment 1. Alerting effect = RT (no cue) – RT (double cue), Orienting effect = RT (center cue) – RT (spatial cue), Executive control effect = RT (incongruent) – RT (congruent). **p < 0.01.

The 2 (modality type: V, AV) × 2 (cue type: no cue, double cue) repeated-measures ANOVA showed that the main effect of the modality type was significant [*F* (1, 43) = 107.662, *p* < 0.001, η_p_^2^ = 0.715]. The responses to the visual stimuli (527.90 ms) were slower than those to the audiovisual stimuli (514.58 ms). The main effect of cue type was also significant [*F* (1, 43) = 289.817, *p* < 0.001, η_p_^2^ = 0.817]. The results showed that the responses in no cue condition (538.08 ms) were slower than those in double cue condition (504.39 ms), which suggested that alerting effect occurred. There was a significant interaction between modality type (V, AV) and cue type (no cue, double cue) [*F* (1, 43) = 11.203, *p* = 0.002, η_p_^2^ = 0.207]. The value of alerting effect under visual condition (*M* = 37.36 ms, *SD* = 15.86 ms) was significant bigger than audiovisual condition (*M* = 30.03 ms, *SD* = 14.10 ms, *t* (43) = 3.347, *p* *=* 0.002, *d* = 0.487, 95% *CI* = [2.92, 11.75]) (see Figure 2).

##### The orienting effect

The 2 (modality type: V, AV) × 2 (cue type: center cue, spatial cue) repeated-measures ANOVA showed that the main effect of the modality type was significant [*F* (1, 43) = 36.847, *p* < 0.001, η_p_^2^ = 0.461]. The responses to the visual stimuli (493.69 ms) were slower than those to the audiovisual stimuli (486.15 ms). The main effect of cue type was also significant [*F* (1, 43) = 172.001, *p* < 0.001, η_p_^2^ = 0.800]. The results showed that the responses in center cue condition (506.78 ms) were slower than those in spatial cue condition (473.06 ms), which suggested that orienting effect occurred. There was a significant interaction between modality type (V, AV) and cue type (center cue, spatial cue) [*F* (1, 43) = 12.954, *p* *=* 0.001, η_p_^2^ = 0.232]. The value of orienting effect under visual condition (*M* = 36.62 ms, *SD* = 17.86 ms) was significant bigger than audiovisual condition (*M* = 30.82 ms, *SD* = 17.88 ms, *t* (43) = 3.599, *p* *=* 0.001, *d* = 0.325, 95% *CI* = [2.55, 9.05]) (see Figure 2).

##### The executive control effect

The 2 (modality type: V, AV) × 2 (flanker type: congruent, incongruent) repeated-measures ANOVA showed that the main effect of the modality type was significant [*F* (1, 43) = 68.872, *p* < 0.001, η_p_^2^ = 0.616]. The responses to the visual stimuli (520.05 ms) were slower than those to the audiovisual stimuli (510.47 ms). The main effect of flanker type was also significant [*F* (1, 43) = 472.840, *p* < 0.001, η_p_^2^ = 0.917]. The results showed that the responses in incongruent condition (544.38 ms) were slower than those in congruent condition (486.14 ms), which suggested that executive control effect occurred. However, there was no significant interaction between modality type and flanker type [*F* (1, 43) = 3.998, *p* *=* 0.052, η_p_^2^ = 0.085]. The value of executive control effect under visual condition (*M* = 60.15 ms, *SD* = 19.99 ms) was analogous to that under audiovisual condition (*M* = 56.34 ms, *SD* = 17.67 ms, *t* (43) = 2.000, *p* *=* 0.052, *d* = 0.201, 95% *CI* = [−0.03, 7.67]) (see Figure 2).

#### Relative Multisensory Response Enhancement (rMRE)

Significant rMRE was observed in all cue conditions as indicated by one-sample t-tests [no cue: *M* = 3.10 ms, *SD* = 2.57 ms, *t* (43) = 8.014, *p* < 0.001, *d* = 1.206; double cue: *M* = 1.54 ms, *SD* = 2.54 ms, *t* (43) = 4.007, *p* < 0.001, *d* = 0.606; center cue: *M* = 1.62 ms, *SD* = 2.76 ms, *t* (43) = 3.893, *p* < 0.001, *d* = 0.587; spatial cue: *M* = 0.85 ms, *SD* = 2.31 ms, *t* (43) = 2.441, *p* = 0.019, *d* = 0.368]. Significant rMRE was observed in all flanker conditions as indicated by one-sample t-tests [neutral: *M* = 2.08 ms, *SD* = 2.21 ms, *t* (43) = 6.225, *p* < 0.001, *d* = 0.941; congruent: *M* = 1.70 ms, *SD* = 2.06 ms, *t* (43) = 5.472, *p* < 0.001, *d* = 0.825; incongruent: *M* = 2.24 ms, *SD* = 1.77 ms, *t* (43) = 8.406, *p* < 0.001, *d* = 1.266].

**Figure 3.**
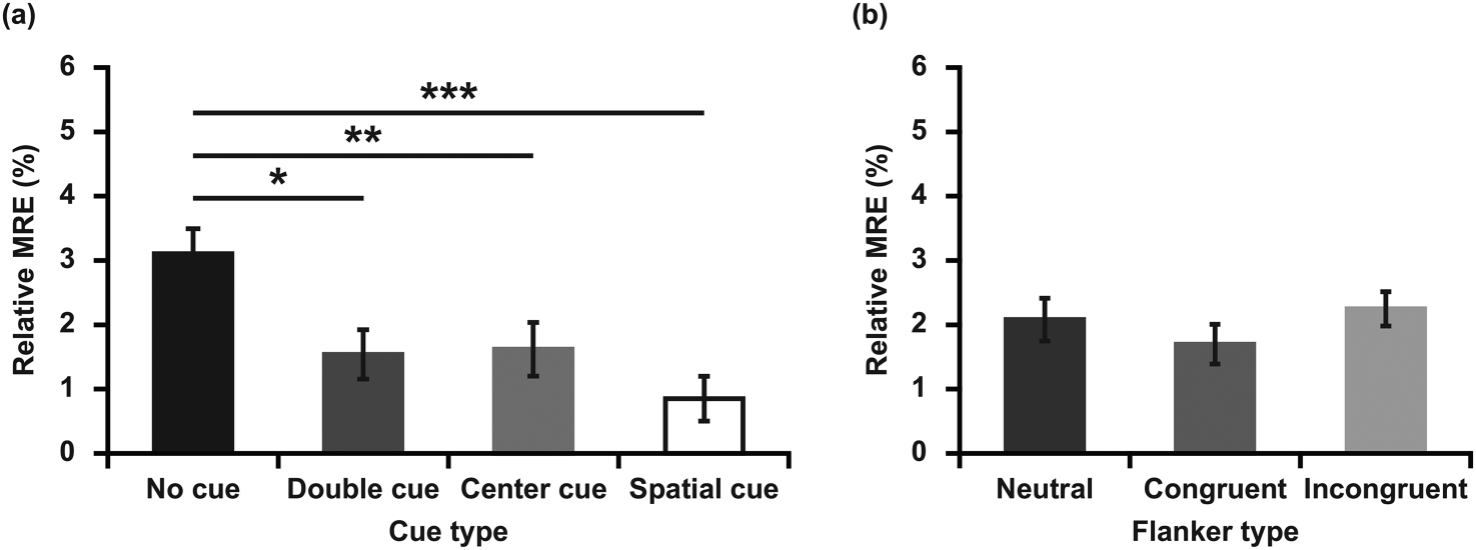
Mean relative multisensory response enhancement (rMRE, %) for each cue type and flanker type in Experiment 1. (a) Magnitude of relative multisensory response enhancement (rMRE, %) in four cue types. (b) Magnitude of relative multisensory response enhancement (rMRE, %) in three flanker types. *p < 0.05, **p < 0.01, ***p < 0.001.

To test for difference in the amount of rMRE, a repeated-measures ANOVA was used. There was a main effect of cue type [*F* (3,129) = 6.926, *p* < 0.001, η_p_^2^ = 0.139]. Planned pairwise comparisons indicated that rMRE was significantly larger in the no cue condition compared to the double cue [*t* (43) = 2.652, *p* = 0.011, *d* = 0.611], the center cue [*t* (43) = 3.071, *p* = 0.004, *d* = 0.554], and the spatial cue conditions [*t* (43) = 4.633, *p* < 0.001, *d* = 0.930]. The main effect of flanker type was not significant [*F* (2, 86) = 0.988, *p* = 0.377, η_p_^2^ = 0.022] (see Figure 3).

### Discussion

In Experiment 1, we investigated whether bilateral auditory stimuli affected the alerting, orienting and executive control networks of visual attention. First, the results of unimodal visual condition replicated the effects of Fan et al. (2002). Second, the results of audiovisual condition revealed significant alerting, orienting and executive control effects. Third, values of alerting and orienting effects in the audiovisual condition were significantly smaller than those in the visual target condition (see Figure 2). No significant difference on the value of executive control effect between audiovisual condition and visual target condition was found (see more discussion in “General Discussion”).

The value of alerting effect was measured by the reaction time difference between no cue and double cue conditions. Furthermore, the value of orienting effect was measured by the reaction time difference between center cue and spatial cue conditions. Smaller reaction time differences for alerting and orienting effects indicated lower efficiency (Johnston et al., 2019). The bilateral auditory stimuli reduced the efficiency of visual alerting and orienting in Experiment 1, which may be caused by the redundant role of cues and bilateral auditory stimuli. Previous studies have demonstrated the alerting and orienting effects were evoked by cues, which could provide temporal information for the upcoming visual target (Correa et al., 2004; Spagna et al., 2015b; Stewart & Amitay, 2015). The alerting and orienting processes could also be triggered by auditory stimuli, which contained temporal information related to the appearance for the visual target (Salmi et al., 2007). The cues in Experiment 1 were not as helpful in alerting and orienting to visual target, since participants could also know when the target would be presented according to the auditory stimuli (Van der Stoep et al., 2015). That is, the role of cues and bilateral auditory stimuli was redundant, which may be the reduced visual alerting and orienting efficiency caused by bilateral auditory stimuli.

We observed the relative multisensory responses enhancement was larger in no cue condition than cue conditions, which is in line with previous studies (Tang et al., 2019; Van der Stoep et al., 2015; Van der Stoep et al., 2017). No cue condition provided no temporal or spatial cue prior to the target. Double and center cues provided temporal information about the imminent appearance of the target. Spatial cue provided both temporal and spatial information, orienting attention to the appropriate location before the target arrives (Fan et al., 2002). In Experiment 1, the amount of relative multisensory responses enhancement was the least at spatial cue condition (see Figure 3). Although the differences between the cues were not significant, it may reflect a trend that the more accurate the information provided by the cues, the lower the multisensory responses enhancement. More reasons about the difference of rMRE under different cue conditions were described in detail in the General Discussion.

Given that the response times by participants might be influenced by the spatial location of the auditory stimuli, Experiment 2 altered the design of Experiment 1. The ipsilateral auditory stimulus was used to ensure that participants could obtain the spatial information related to the direction of the central arrow in Experiment 2. Note that the ipsilateral auditory stimulus could accurately direct the direction of the target arrow.

## Experiment 2

### Materials and Methods

#### Participants

Forty-eight healthy participants took part in the experiment and one participant was excluded for failing to attain 80% on accuracy rate. The final sample consisted of forty-seven undergraduate University students aged 19–27 years (34 females, *M* = 22.55 years, *SD* = 2.47). The sample size calculated using G*Power toolbox was the same as Experiment 1. All participants reported normal or corrected-to-normal vision, no hearing impairment, no reported history of attention deficit disorder, and no history of brain damage. Before the experiment, all participants gave written informed consent. The study was conducted according to the ethical guidelines of the Liaoning Normal University Ethics Committee.

#### Apparatus, Stimuli, Procedure and Design

In Experiment 2, the apparatus, visual stimuli, procedures, and data analysis were the same as Experiment 1. The only difference was that the auditory stimulus in Experiment 2 was presented by one peripheral speaker on the left or right side of the screen (see Figure 1). Note that the location of the auditory stimulus was the same as the direction of the central arrow, suggesting that the ipsilateral auditory stimulus could accurately direct the direction of target arrow. All participants reported being able to identify the location of the sound. Participants were instructed to identify the direction (left or right) of the central arrow and ignore the other flanker arrows or lines, as quickly and accurately as possible, using two keys (“F” for the left, and “J” for the right) on the keyboard.

#### Data Analysis

In Experiment 2, the data analysis method was the same as that in Experiment 1. All missing or false trials were removed from the data. Outliers were defined as three standard deviations (SD) above or below the mean reaction times, and were removed from the experimental data. In Experiment 2, the final data deletion accounted for 3.08% of the total data.

### Results

#### Accuracy

Accuracy was high with participants completing above 90% of trials correctly overall. A repeated 4 × 3 × 2 ANOVA with cue type (no cue, double cue, center cue, and spatial cue), flanker type (neutral, congruent, and incongruent) and modality type (V, AV) was conducted on accuracy. There was a main effect of cue type [*F* (3, 138) = 8.744, *p* < 0.001, η_p_^2^ = 0.160], which was driven by participants responding more accurately to spatial cue (98.65%) than center cue (97.54%, *p* < 0.001). The main effect of flanker type was significant [*F* (2, 92) = 40.893, *p* < 0.001, η_p_^2^ = 0.471], caused by participants responding more accurately to congruent condition (99.45%) than neutral condition (99.04%, *p* = 0.001) or incongruent condition (95.97%, *p* < 0.001). There was a main effect of modality type [*F* (1, 46) = 6.471, *p* = 0.014, η_p_^2^ = 0.123], indicating that participants responding more accurately in audiovisual modality compared to visual modality (98.42% vs. 97.89%). A significant modality type and flanker type interaction [*F* (2, 92) = 3.898, *p* *=* 0.047*,* η_p_^2^ = 0.078] and a significant cue type and flanker type interaction [*F* (6, 276) = 7.256, *p* < 0.001*,* η_p_^2^ = 0.136] were revealed. No significant interaction was found in modality type and cue type [*F* (3, 138) = 0.867, *p* *=* 0.460, η_p_^2^ = 0.018]. The three-way interaction between modality type, cue type, and flanker type was not significant [*F* (6, 276) = 1.978, *p* *=* 0.101, η_p_^2^ = 0.041].

#### Reaction Times

A repeated 4 × 3 × 2 ANOVA with cue type (no cue, double cue, center cue, and spatial cue), flanker type (neutral, congruent, and incongruent), and modality type (V, AV) was conducted on reaction times. There was a main effect of cue type [*F* (3, 138) = 91.935, *p* < 0.001, η_p_^2^ = 0.667], indicating that participants responded more slowly in no cue condition (497.13 ms) than in double cue (482.73 ms, *p* < 0.001), center cue (482.72 ms, *p* < 0.001), and spatial cue conditions (462.64 ms, *p* < 0.001). In addition, participants responded more slowly in both center cue and double cue conditions than in spatial cue condition (center cue vs. spatial cue, *p* < 0.001; double cue vs. spatial cue, *p* < 0.001). There was a main effect of flanker type [*F* (2, 92) = 386.432, *p* < 0.001, η_p_^2^ = 0.894], caused by participants responding more slowly in the case of incongruent condition (522.39 ms) than in the case of neutral (461.81 ms, *p* < 0.001) and congruent conditions (459.72 ms, *p* < 0.001). The main effect of modality type was significant [*F* (1, 46) = 33.468, *p* < 0.001, η_p_^2^ = 0.421], which was driven by participants responding more slowly to visual modality than audiovisual modality (507.09 ms vs. 455.52 ms).

The interaction between modality type and flanker type was significant [*F* (2, 92) = 6.963, *p* = 0.006, η_p_^2^ = 0.131], which could be explained by differences in the sizes of flanker effects for different modalities. A significant cue type and flanker type interaction [*F* (6, 276) = 6.207, *p* < 0.001, η_p_^2^ = 0.119] was revealed, and a three-way interaction between cue type, flanker type, and modality type was significant [*F* (6, 276) = 4.011, *p* = 0.001, η_p_^2^ = 0.080]. However, the interaction between modality type and cue type was not significant [*F* (3, 138) = 2.145, *p* = 0.097, η_p_^2^ = 0.045].

#### Attention Network Effects

We reported the difference of attention network effects between visual and audiovisual modalities using repeated-measures ANOVA.

##### The alerting effect

**Figure 4.**
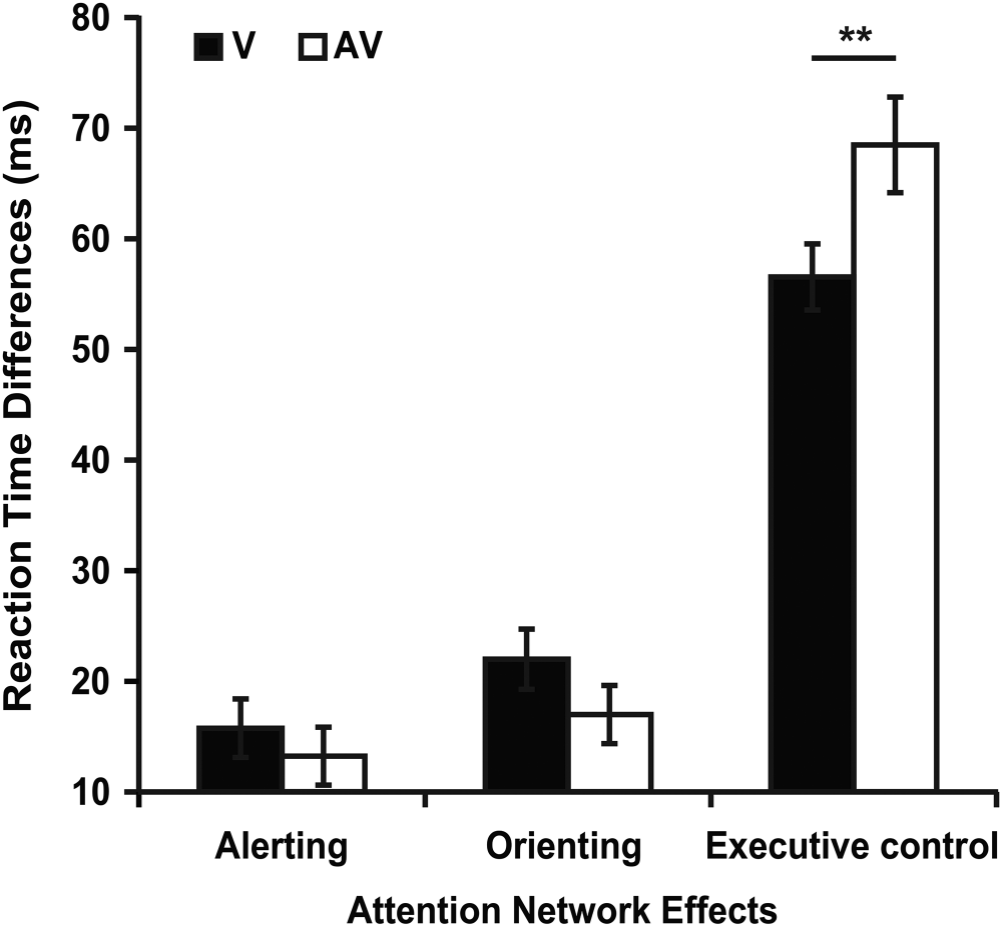
The reaction time differences of attention network effects between visual and audiovisual modalities in Experiment 2. Alerting effect = RT (no cue) – RT (double cue), Orienting effect = RT (center cue) – RT (spatial cue), Executive control effect = RT (incongruent) – RT (congruent). **p < 0.01.

The 2 (modality type: V, AV) × 2 (cue type: no cue, double cue) repeated-measures ANOVA showed that the main effect of the modality type was significant [*F* (1, 46) = 36.871, *p* < 0.001, η_p_^2^ = 0.445]. The responses to the visual stimuli (516.15 ms) were slower than those to the audiovisual stimuli (462.40 ms). The main effect of cue type was also significant [*F* (1, 46) = 61.738, *p* < 0.001, η_p_^2^ = 0.573]. The results showed that the responses in no cue condition (496.52 ms) were slower than those in double cue condition (482.03 ms), which suggested that alerting effect occurred. However, there was no significant interaction between modality type (V, AV) and cue type (no cue, double cue) [*F* (1, 46) = 0.450, *p* = 0.506, η_p_^2^ = 0.010]. The value of alerting effect under visual condition (*M* = 15.75 ms, *SD* = 18.16 ms) was analogous to that under audiovisual condition (*M* = 13.22 ms, *SD* = 17.97 ms, *t* (46) = 0.671, *p* *=* 0.506, *d* = 0.140, 95% *CI* = [−5.06, 10.11]) (see Figure 4).

##### The orienting effect

The 2 (modality type: V, AV) × 2 (cue type: center cue, spatial cue) repeated-measures ANOVA showed that the main effect of the modality type was significant [*F* (1, 46) = 29.629, *p* < 0.001, η_p_^2^ = 0.392]. The responses to the visual stimuli (496.51 ms) were slower than those to the audiovisual stimuli (477.45 ms). The main effect of cue type was also significant [*F* (1, 46) = 78.271, *p* < 0.001, η_p_^2^ = 0.630]. The results showed that the responses in center cue condition (481.72 ms) were slower than those in spatial cue condition (462.23 ms), which suggested that orienting effect occurred. However, there was no significant interaction between modality type (V, AV) and cue type (center cue, spatial cue) [*F* (1, 46) = 2.725, *p* = 0.106, η_p_^2^ = 0.056]. The value of orienting effect under visual condition (*M* = 21.99 ms, *SD* = 18.65 ms) was analogous to that under audiovisual condition (*M* = 16.98 ms, *SD* = 18.01 ms, *t* (46) = 1.651, *p* *=* 0.106, *d* = 0.273, 95% *CI* = [−1.10, 11.11]) (see Figure 4).

##### The executive control effect

The 2 (modality type: V, AV) × 2 (flanker type: congruent, incongruent) repeated-measures ANOVA showed that the main effect of the modality type was significant [*F* (1, 46) = 28.021, *p* < 0.001, η_p_^2^ = 0.379]. The responses to the visual stimuli (515.88 ms) were slower than those to the audiovisual stimuli (466.01 ms). The main effect of flanker type was also significant [*F* (1, 46) = 416.924, *p* < 0.001, η_p_^2^ = 0.901]. The results showed that the responses in incongruent condition (522.19 ms) were slower than those in congruent condition (459.69 ms), which suggested that executive control effect occurred. There was a significant interaction between modality type and flanker type [*F* (1, 46) = 8.024, *p* *=* 0.007, η_p_^2^ = 0.149]. The value of executive control effect under audiovisual condition (*M* = 68.48 ms, *SD* = 29.64 ms) was significant bigger than visual condition (*M* = 56.54 ms, *SD* = 20.49 ms, *t* (46) = 2.833, *p* *=* 0.007, *d* = 0.454, 95% *CI* = [3.46, 20.43]) (see Figure 4).

#### Relative Multisensory Response Enhancement (rMRE)

Significant rMRE was observed in all cue conditions as indicated by one-sample t-tests [no cue: *M* = 10.50 ms, *SD* = 10.95 ms, *t* (46) = 6.574, *p* < 0.001, *d* = 0.959; double cue: *M* = 10.61 ms, *SD* = 13.18 ms, *t* (46) = 5.520, *p* < 0.001, *d* = 0.805; center cue: *M* = 10.31 ms, *SD* = 12.85 ms, *t* (46) = 5.502, *p* < 0.001, *d* = 0.802; spatial cue: *M* = 9.39 ms, *SD* = 12.58 ms, *t* (46) = 5.114, *p* < 0.001, *d* = 0.746]. Significant rMRE was observed in all flanker conditions as indicated by one-sample t-tests [neutral: *M* = 10.57 ms, *SD* = 10.76 ms, *t* (46) = 6.732, *p* < 0.001, *d* = 0.982; congruent: *M* = 10.32 ms, *SD* = 11.21 ms, *t* (46) = 6.314, *p* < 0.001, *d* = 0.921; incongruent: *M* = 7.36 ms, *SD* = 14.51 ms, *t* (46) = 3.478, *p* = 0.001, *d* = 0.507].

**Figure 5.**
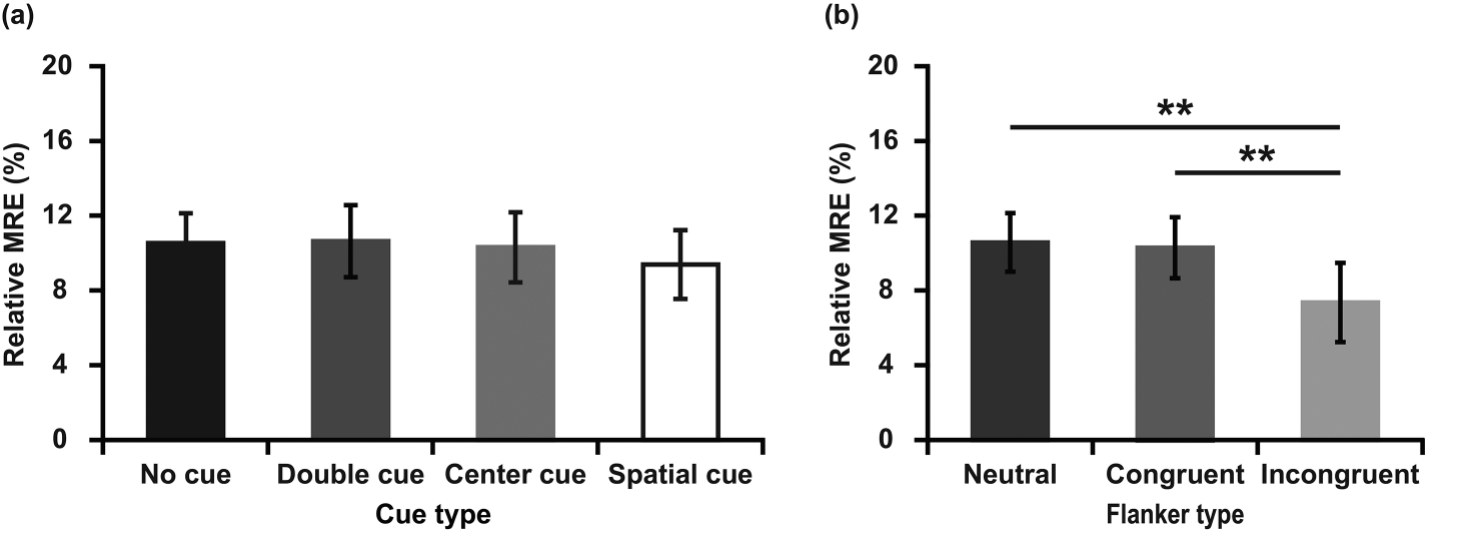
Mean relative multisensory response enhancement (rMRE, %) for each cue type and flanker type in Experiment 2. (a) Magnitude of relative multisensory response enhancement (rMRE, %) in four cue types. (b) Magnitude of relative multisensory response enhancement (rMRE, %) in three flanker types. **p < 0.01.

To test for difference in the amount of rMRE, a repeated-measures ANOVA was used. The main effect of cue type was not significant [*F* (3,138) = 1.266, *p* = 0.289, η_p_^2^ = 0.027]. There was a main effect of flanker type [*F* (2, 92) = 9.982, *p* = 0.001, η_p_^2^ = 0.178]. Planned pairwise comparisons indicated that rMRE was significantly smaller in the incongruent condition compared to the neutral condition [*t* (46) = 3.670, *p* = 0.001, *d* = 0.246], and the congruent condition [*t* (46) = 2.993, *p* = 0.004, *d* = 0.225]. (Figure 5)

### Discussion

The aim of Experiment 2 was to confirm whether ipsilateral auditory stimulus affected the alerting, orienting and executive control networks of visual attention. First, the significant alerting, orienting and executive control effects were found in both visual and audiovisual conditions, which is consistent with the results of Experiment 1. Second, values of alerting and orienting effects in the audiovisual condition were not significantly different from those in the visual target condition (see more discussion in “General Discussion”). Third, value of executive control effect in the audiovisual condition was significantly larger than those in the visual target condition (see Figure 4). The value of executive control effect was measured by the reaction time difference between incongruent and congruent conditions. Larger reaction time difference (i.e., value of executive control effect), lower efficiency for executive control effect (Macleod et al., 2010; Posner, 2008; Weinbach & Henik, 2013). That is, the efficiency of executive control effect in the audiovisual condition was significantly smaller than those in the visual target condition in Experiment 2.

No matter with or without accompanying sound, the reaction times were significantly longer at incongruent flanker condition than congruent or neutral flanker condition. While there was no significant difference between neutral flanker and congruent flanker conditions (Salagovic & Leonard, 2021). It suggested that executive control effect was often largely due to interference from incongruent flankers, but not facilitation from congruent flankers (Salagovic & Leonard, 2021; Schaffer & La Berge, 1979). We argued that the ipsilateral auditory stimulus reduced the efficiency of visual executive control, which may be caused by two reasons. First, the study adopted the ANT paradigm, but its results were consistent with the results of the flanker tasks studied in previous study (Fong et al., 2018). The responses to the visual target with congruent sound were significantly faster than responses to the unimodal visual target, while the target with incongruent sound were significantly slower than the unimodal visual target. This leaded to a larger difference in response time between incongruent and congruent sound conditions in audiovisual condition. Larger reaction time difference, lower efficiency for executive control effect. Second, in Experiment 2, the auditory stimuli were set to be spatially predictive. Participants were instructed to resolve not only visual flanker conflicts, but also the conflict between cross-modality auditory stimuli and incongruent flankers. In other words, the flanker conflicts were enhanced in audiovisual condition. When we faced dual-conflict, concurrent another conflict processes might arise interference, negatively impacting our performance (Spagna et al., 2020). We thus observed the decrease in efficiency of executive control effect when the auditory stimuli interfered with flankers.

## General Discussion

The primary goal of this study was to investigate the effects of bilateral and ipsilateral auditory stimuli on the subcomponents of visual attention. The results showed the bilateral auditory stimuli reduced the efficiency of visual alerting and orienting, but had no significant effect on the efficiency of visual executive control (Experiment 1). The ipsilateral auditory stimulus reduced the efficiency of visual executive control, but had no significant effect on the efficiency of visual alerting and orienting (Experiment 2). The results of Experiment 1 also showed a larger rMRE (i.e., larger multisensory integration effect) at no cue condition compared with cue conditions. While, the results of Experiment 2 showed a larger rMRE at congruent condition compared with incongruent condition. Taken together, the bilateral and ipsilateral auditory stimuli have different effects on the subcomponents of visual attention.

### The Effect of Bilateral or Ipsilateral Auditory Stimuli on Visual Attention Network

Participants were instructed to distinguish the direction of visual target arrow in both Experiment 1 and Experiment 2. The difference between two experiments was whether the auditory stimuli contained spatial information associated with the direction of visual target arrow. As we known, temporally synchronized auditory stimuli can facilitate participants’ performance on visual tasks (Van der Burg et al., 2008). Making auditory stimuli spatially informative with regard to the target location can reduce reaction times still further (Ngo & Spence, 2010).

The bilateral auditory stimuli provided only temporal information related to the presentation for the visual target (Gao et al., 2014) and cues could provide temporal information about the visual target as well in Experiment 1. We argued that the role of bilateral auditory stimuli and cues was redundant, and the bilateral auditory stimuli reduced the efficiency of visual alerting and orienting, which has been discussed in “2.3 Discussion”. Nevertheless, the bilateral auditory stimuli did not provide any spatial information associate with visual target, which had no help on the inhibition of flankers and the discrimination of arrow direction. Thus, the bilateral auditory stimuli had no effect on the efficiency of visual executive control, which is in line with our hypothesis.

Unlike bilateral auditory stimuli, ipsilateral auditory stimulus provided not only temporal information for the appearance of visual target, but also spatial information consistent with the direction of the target arrow (Gao et al., 2014). In Experiment 2, all cues could provide temporal information and evoking the alerting and orienting effects (Correa et al., 2004; Spagna et al., 2015b; Stewart & Amitay, 2015). The ipsilateral auditory stimulus played a more important role in affecting the discrimination of arrow direction, which was irreplaceable by cues. That is, the role of ipsilateral auditory stimulus and cues was not redundant, and there was no significant difference in the sizes of cue effects for different modalities. Thus, the ipsilateral auditory stimulus had no effect on the visual alerting and orienting. It was also possible that the interaction between cues and flankers leaded to the above results. In the ANT paradigm, the attention networks are not completely independent but interconnected (Schneider, 2019; Spagna et al., 2015b). There may be the ipsilateral auditory stimulus has too much influence on the visual executive control, so that it overwhelms the influence on alerting and orienting. Future work is required to focus on investigating the interaction between attention networks in the audiovisual condition. Moreover, the spatial information provided by the ipsilateral auditory stimulus might influence the discrimination of arrow direction, thereby affecting the efficiency of visual executive control (see more in “3.3 Discussion”). However, there are still deficiencies in this study. For example, the ipsilateral auditory stimulus was 100% predictable of the visual target direction in Experiment 2. The participants might simply use the auditory stimulus to make their decisions in audiovisual condition. To make better conclusions, one requires the presentation of unimodal auditory trials as No-go trials.

### Relative Multisensory Response Enhancement: Cue vs. No cue Condition, Incongruent vs. Congruent Condition

Results from Experiment 1 found that relative multisensory response enhancement was decreased at cue conditions compared with no cue condition, but there was no difference in all flanker conditions. Van der Stoep et al. (2015) proposed two hypotheses to explain this reduction at cue condition: spatial uncertainty of target location and perceptual sensitivity. First, higher uncertainty of the target location, higher need for spatial orienting (Van der Stoep et al., 2015). When the spatial orienting evoked by the cues and the spatial orienting caused by the synchronized auditory stimuli is redundant, the multisensory integration was not as helpful since attention has already oriented to the peripheral location by cues (Tang et al., 2019). Second, compared with no cue condition, the perceptual sensitivity was increased at cue conditions (Carrasco, 2011; Carrasco et al., 2008). Studies have shown that the multisensory integration process followed the principle of inverse effectiveness, the benefit of multisensory integration is larger for weaker stimuli than for stronger stimuli (Otto et al., 2013; Senkowski et al., 2011; Tang et al., 2019). Therefore, multisensory integration was reduced at cue conditions.

Results from Experiment 2 found that relative multisensory response enhancement was decreased at incongruent condition compared with congruent condition, but there was no difference in all cue conditions. The multisensory stimuli congruence is a critical factor in multisensory behavioral performance. Previous studies have shown that incongruent stimuli have an effect on the modulation of multisensory integration (Guo et al., 2015; Laurienti et al., 2004). Li et al. (2020) used unisensory stimuli (animal images or sounds) and multisensory stimuli (semantically congruent audiovisual objects or semantically incongruent audiovisual objects) to assess multisensory integration. Results indicated that semantically incongruent animal sounds and images were not integrated (Li et al., 2020). More than that, the incongruent flankers conflicted with not only target arrow but also auditory stimuli in Experiment 2. Participants were struggled to cope with dual-conflict, which could weaken the multisensory integration process in the audiovisual condition.

Investigating the alerting, orienting and executive control of attention networks can benefit us to understand attentional deficits and psychiatric disorders. For example, Spagna et al. (2015a) found that compared with healthy controls, patients with schizophrenia had poor efficiency in all three attention network functions. While the orienting function was improved post-treatment in patients with schizophrenia, there was no evidence for improvement in the alerting and executive control (Spagna et al., 2015a). In addition, Elliott et al. (2014) examined the influence of meditation training on three attention network functions. Results suggested that meditation training improved both alerting and executive control, but not orienting (Elliott et al., 2014). These studies indicated that the efficiency of attention network functions could be improved by treatment and training for patients with neurological and psychiatric disorders. In this study, we found that when the visual target was accompanied by a congruent sound stimulus, the participants responded faster and more accurately. In order to more effectively improve the ability to resolve conflicts for patients, congruent multisensory information can be added to psychological interventions.

In summary, the current results clearly provide the first empirical evidence for the alerting, orienting and executive control effects in audiovisual condition. Both the bilateral and ipsilateral auditory stimuli affect the efficiency of visual attention networks, but the affected subcomponents are different. The bilateral auditory stimuli provide temporal information related to the presentation for visual target, which weaken the role of cues and affect visual alerting and orienting efficiency specifically. The ipsilateral auditory stimulus provides not only temporal information but also spatial information consistent with the direction of the target arrow, which affects the executive control effect. Specifically, the ipsilateral auditory stimulus increases the difference in response time between congruent and incongruent conditions. We also found a reduced rMRE effect at cue conditions compared with no cue condition, and a larger rMRE effect at congruent condition compared with incongruent condition. Finally, the results help to further illuminate how we resolve the conflicts of multisensory scenes in our daily life.
